# The Aryl Hydrocarbon Receptor Ligand FICZ Improves Left Ventricular Remodeling and Cardiac Function at the Onset of Pressure Overload-Induced Heart Failure in Mice

**DOI:** 10.3390/ijms23105403

**Published:** 2022-05-12

**Authors:** María Tamayo, Laura Martín-Nunes, María José Piedras, María Martin-Calvo, Daniel Martí-Morente, Marta Gil-Fernández, Nieves Gómez-Hurtado, María Ángeles Moro, Lisardo Bosca, María Fernández-Velasco, Carmen Delgado

**Affiliations:** 1 Instituto de Investigaciones Biomédicas “Alberto Sols” (CSIC-UAM), CIBER de Enfermedades Cardiovasculares (CIBERCV), 28029 Madrid, Spain; mtamayo@iata.csic.es (M.T.); lauramartin@iib.uam.es (L.M.-N.); mj.garcia.prof@ufv.es (M.J.P.); mmcalvo@iib.uam.es (M.M.-C.); danielmarti7@hotmail.com (D.M.-M.); martagil@iib.uam.es (M.G.-F.); nievesgh@hotmail.com (N.G.-H.); lbosca@iib.uam.es (L.B.); mvelasco@iib.uam.es (M.F.-V.); 2 Facultad de Medicina, Universidad Francisco de Vitoria (UFV), 28223 Madrid, Spain; 3 Innate Immune Response Group, IdiPAZ, La Paz University Hospital, 28046 Madrid, Spain; 4 Centro Nacional de Investigaciones Cardiovasculares (CNIC), Melchor Fernández Almagro 3, 28029 Madrid, Spain; mariaangeles.moro@cnic.es

**Keywords:** aryl hydrocarbon receptor (AhR), fibrosis, cardiac hypertrophy, oxidative stress, cardiac remodeling, formyl-indolo [3,2-b] carbazole (FICZ)

## Abstract

Adverse ventricular remodeling is the heart’s response to damaging stimuli and is linked to heart failure and poor prognosis. Formyl-indolo [3,2-b] carbazole (FICZ) is an endogenous ligand for the aryl hydrocarbon receptor (AhR), through which it exerts pleiotropic effects including protection against inflammation, fibrosis, and oxidative stress. We evaluated the effect of AhR activation by FICZ on the adverse ventricular remodeling that occurs in the early phase of pressure overload in the murine heart induced by transverse aortic constriction (TAC). Cardiac structure and function were evaluated by cardiac magnetic resonance imaging (CMRI) before and 3 days after Sham or TAC surgery in mice treated with FICZ or with vehicle, and cardiac tissue was used for biochemical studies. CMRI analysis revealed that FICZ improved cardiac function and attenuated cardiac hypertrophy. These beneficial effects involved the inhibition of the hypertrophic calcineurin/NFAT pathway, transcriptional reduction in pro-fibrotic genes, and antioxidant effects mediated by the NRF2/NQO1 pathway. Overall, our findings provide new insight into the role of cardiac AhR signaling in the injured heart.

## 1. Introduction

Adverse cardiac remodeling is a mechanism of regional or global structural and functional changes in the heart as a consequence of damaging stimuli, such as myocardial infarction, pressure overload (aortic stenosis, hypertension), inflammatory heart muscle disease (myocarditis), idiopathic dilated cardiomyopathy, or volume overload (valvular regurgitation) [1,2]. Cardiac remodeling is an important determinant of the clinical outcome of heart failure (HF) and is linked to disease progression and poor prognosis. The remodeling process is characterized by profuse inflammatory and pro-fibrotic responses, neurohormonal activation, elevated production of reactive oxygen species (ROS), and heart enlargement, in an effort to manage increases in hemodynamic demand [3,4,5]. Although heart enlargement is initially a compensatory mechanism, sustained hypertrophy combined with fibrosis can ultimately lead to a decline in left ventricular function, which is an independent risk factor for HF [6].

The aryl hydrocarbon receptor (AhR) is a ligand-activated transcription factor of the basic Helix-Loop-Helix (bHLH)-PAS (Per-Arnt-Sim) superfamily that was initially recognized as the mediator of the toxicity of several exogenous (environmental) pollutants, such as 2,3,7,8-tetrachlorodibenzo-p-dioxin (TCDD), dibenzofurans, and related halogenated biphenyls [7,8,9]. In addition to its role in metabolizing xenobiotic compounds as part of an adaptive chemical response, there is robust evidence that AhR has physiological functions and has endogenous ligands. Indeed, AhR is involved in the control of vascular homeostasis and immune system function [10,11]. In addition, early studies on the expression of AhR in different organ systems showed that AhR was significantly expressed in the heart [9]. In the absence of ligand, AhR exists primarily in the cytoplasm complexed with two 90-kDa heat-shock protein chaperones and the AhR inhibitory protein. In the canonical genomic signaling pathway, ligand binding to AhR triggers conformational changes, translocation to the nucleus, and dissociation of the chaperone complex. In the nucleus, AhR associates with its partner protein aryl hydrocarbon receptor nuclear translocator (ARNT) and binds to xenobiotic response DNA elements, which are located in the promoter region of a number of receptor-regulated genes, such as *Cyp1a1*, *Cyp1a2*, *Cyp1b1*, *yp2s1*, *Ahrr*, *Nqo1*, and *Gsta1* [12].

The endogenous function of AhR-signaling pathways related to cardiovascular development and/or homeostasis has been examined in AhR-knockout mice [13]. These animals exhibit age-related cardiovascular alterations, including hypertrophic cardiomyopathy and focal fibrosis [13,14], suggesting that AhR signaling is required for normal heart development and maturation. Along this line, several endogenous AhR ligands have been identified that can contribute to AhR-dependent activity in the heart, including heme metabolites such as bilirubin, or tryptophan metabolites from the monoamine oxidase pathway such as formyl-indolo [3,2-b] carbazole (FICZ) [15] and kynurenine [16,17]. However, the physiological and/or toxicological consequences of AhR activation by endogenous ligands in the context of cardiac damage are not well known. Furthermore, while much attention has been paid to long-term adverse cardiac remodeling in HF, both in clinical practice and in experimental models, less is known about the molecular mechanisms involved at the onset of HF and whether modulation of the acute phase of HF might determine disease progression.

In the present study, we have investigated whether the AhR ligand FICZ can alleviate the adverse cardiac remodeling that occurs during the initial phase of pressure overload induced by transverse aortic constriction (TAC). Our results show that FICZ treatment has a cardioprotective effect in mice, preventing cardiac hypertrophy development, reducing the transcriptional expression of pro-fibrotic genes, and inducing antioxidant effects mediated by increased expression of the *Nrf2/Nq_O_1* pathway.

## 2. Results

### 2.1. FICZ Activates the Aryl Hydrocarbon Receptor Pathway

**Figure 1:**
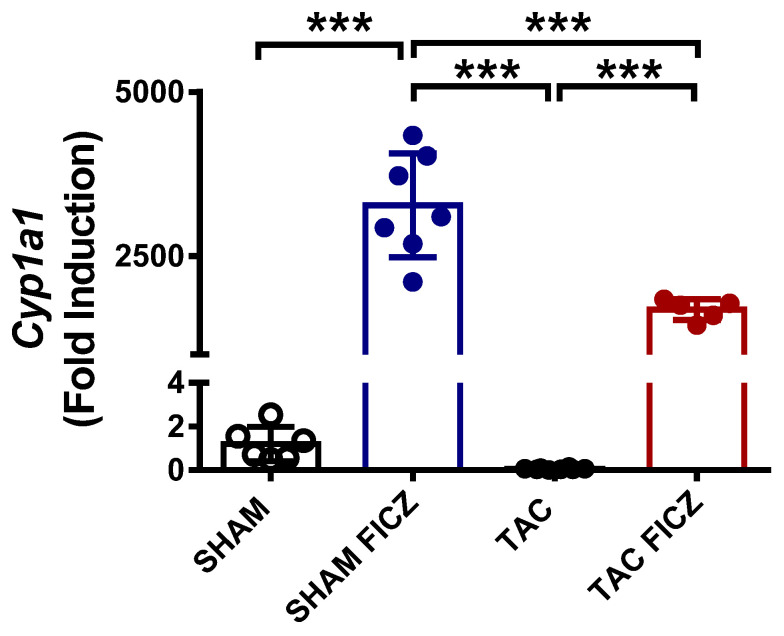
Formyl-indolo [3,2-b] carbazole (FICZ) treatment increases the expression of the aryl hydrocarbon receptor target gene *Cyp1a1* in heart tissue, evaluated by real-time PCR analysis. Groups are: Sham (open circles) (*N* = 6), Sham FICZ (blue circles) (*N* = 7), transverse aortic constriction (TAC) (black circles) (*N* = 8) and TAC FICZ (red circles) (*N* = 5). Data are expressed as mean ± SD. *** *p* < 0.001.

To demonstrate that FICZ activates AhR, we measured the expression of the AhR target gene *Cyp1a1*, encoding cytochrome P450 1A1, in heart tissue of mice treated or not with the ligand (see Methods). As shown in Figure 1, *Cyp1a1* expression was significantly higher in FICZ-treated groups than in vehicle-treated groups. Notably, *Cyp1a1* expression was significantly lower in the FICZ-treated group subjected to TAC than in the FICZ-treated Sham surgery group. 

### 2.2. FICZ Attenuates Cardiac Dysfunction and Prevents Cardiac Hypertrophy at the Onset of Pressure Overload

**Figure 2:**
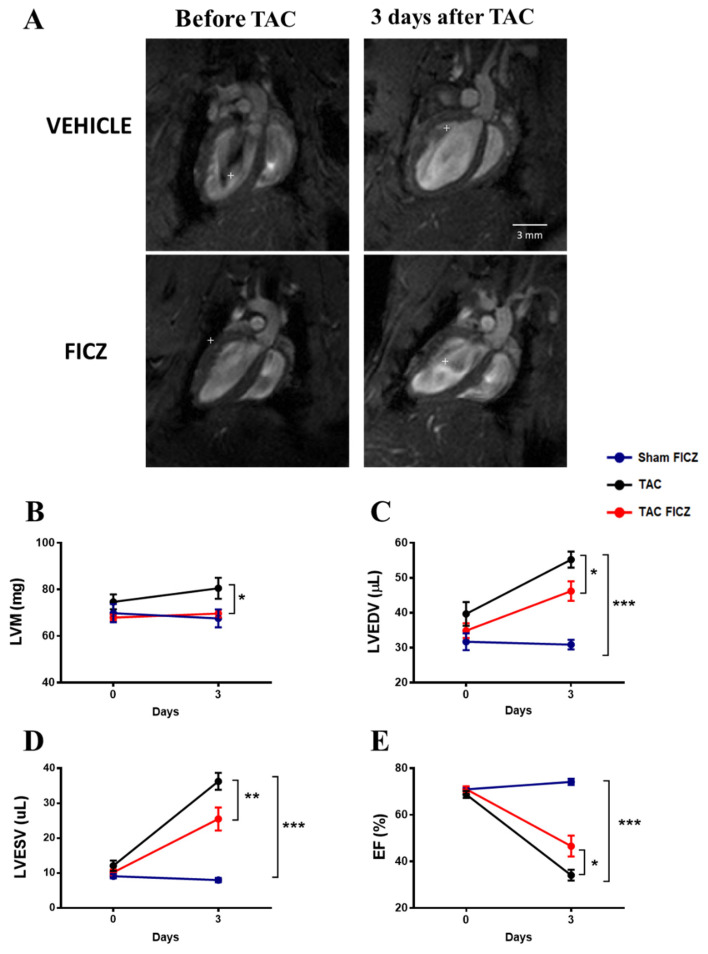
FICZ administration prevents the increase in left ventricular mass (LVM) and improves cardiac dysfunction in mice subjected to TAC surgery: (**A**) Representative cardiac magnetic resonance images (CMRI) of mouse hearts before TAC and 3 days after TAC surgery, and treated with vehicle or with FICZ. CMRI analysis of LVM (**B**), left ventricular end-diastolic volume (LVEDV) (**C**), left ventricular end-systolic volume (LVESV) (**D**), and ejection fraction (EF) (**E**). Groups are: Sham FICZ (blue circles) (*N* = 8), TAC (black circles) (*N* = 8) and TAC FICZ (red circles) (*N* = 8). Data are expressed as mean ± SEM. * *p* < 0.05; ** *p* < 0.01; *** *p* < 0.001.

We next evaluated cardiac structure and function using cardiac magnetic resonance imaging (CMRI) in mice before and 3 days after TAC surgery. Representative CMRI images of four-chamber long-axis views of hearts are shown in Figure 2A. As expected, no changes were observed in left ventricular mass (LVM) 3 days after Sham surgery in the group treated with FICZ (Figure 2B). Analysis of mice subjected to TAC surgery showed that LVM was significantly greater in the group treated with vehicle than in the group treated with FICZ (Figure 2B). Quantification of the CMRI parameters for left ventricular end-diastolic volume (LVEDV) and left ventricular end-systolic volume (LVESV) are shown in Figure 2C,D. Both parameters were unchanged by FICZ administration in mice subjected to Sham surgery, but were significantly increased 3 days after TAC surgery, indicating left ventricle dilation; FICZ administration significantly slowed the progression of dilation. Consistent with these changes, the ejection fraction (EF) was significantly higher in the TAC surgery group treated with FICZ than in equivalent mice treated with vehicle (Figure 2E).

Overall, these data demonstrate that AhR activation by FICZ in the acute phase of pressure overload protects mice from adverse structural and functional cardiac remodeling.

### 2.3. The Antihypertrophic Effect of FICZ Involves the Attenuation of the Calcineurin/NFAT Signaling Pathway

**Figure 3:**
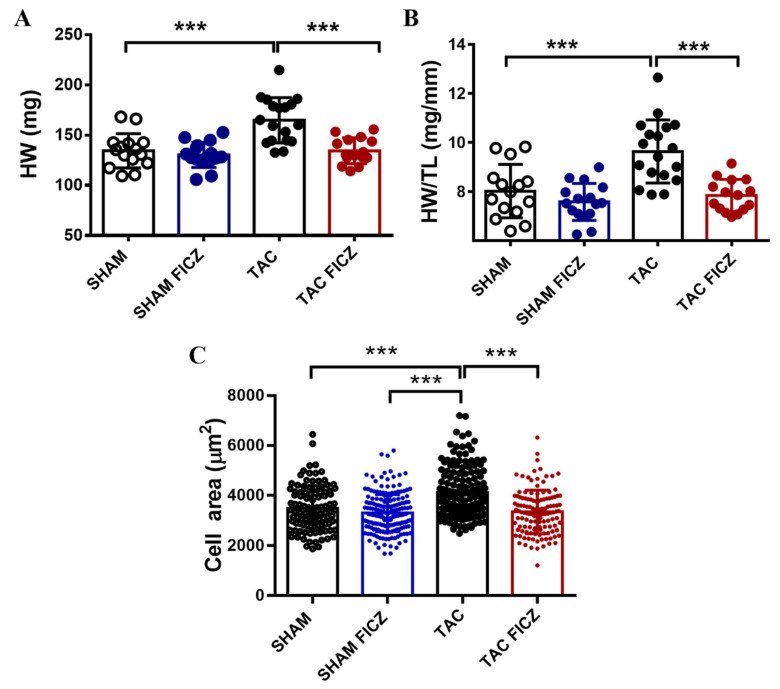
FICZ treatment prevents cardiac hypertrophy induced by TAC: (**A**). Heart weight (HW) (mg). (**B**). Heart Weight/Tibia Length HW/TL (mg/mm). Groups are: Sham (open circles) (*N* = 15), Sham FICZ (blue circles) (*N* = 16), TAC (black circles) (*N* = 18) and TAC FICZ (red circles) (*N* = 15). (**C**). Cardiomyocyte area (µm^2^). Groups are: Sham (open circles) (*n* = 106; *N* = 5), Sham FICZ (blue circles) (*n* = 200; *N* = 4), TAC (black circles) (*n* = 148; *N* = 5) and TAC FICZ (red circles) (*n* = 126; *N* = 4). Data are expressed as mean ± SD. *** *p* < 0.001. *N* = number of mice; *n* = number of cardiomyocytes.

Postmortem assessment of cardiac hypertrophy showed that both the heart weight (HW) (Figure 3A) and the heart-weight/tibia-length (HW/TL) ratio (Figure 3B) were significantly higher in the TAC group than in the Sham group, and FICZ administration suppressed the increase in both parameters in the TAC group. In addition, quantitative analysis of the cardiomyocyte surface area in the four groups showed that cells from the TAC-vehicle group were significantly larger than those from the Sham groups and the TAC-induced increase in size was blocked by FICZ treatment (Figure 3C).

**Figure 4:**
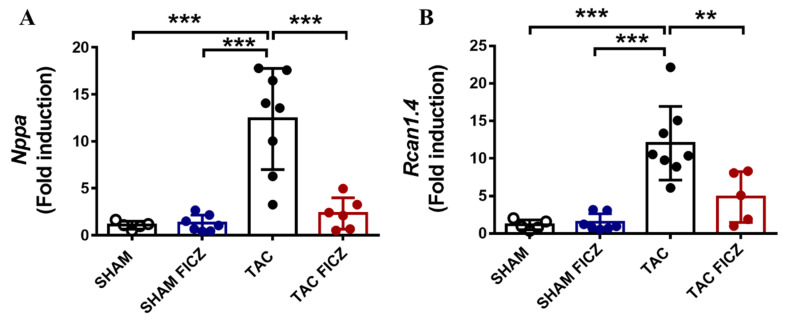
FICZ treatment prevents hypertrophic development by attenuating the calcineurin/RCAN1.4 pathway in mice subjected to TAC surgery. Real-time PCR analysis of mRNA expression of the cardiac hypertrophy biomarker atrial natriuretic peptide (*Nppa*) (**A**) and *Rcan1.4* (**B**). Expression was normalized to that of the *Rplp0* housekeeping gene. Groups are: Sham (open circles) (*N* = 5), Sham FICZ (blue circles) (*N* = 7), TAC (black circles) (*N* = 8) and TAC FICZ (red circles) (*N* = 6). Data are expressed as mean ± SD. ** *p* < 0.01; *** *p* < 0.001.

To gain insight into the molecular mechanisms involved in the antihypertrophic effect of FICZ, we first analyzed the expression of the hypertrophic marker atrial natriuretic peptide (*Nppa*). Results showed that *Nppa* expression was significantly higher in the TAC group than in the Sham group, and treatment with FICZ significantly decreased *Nppa* expression in the TAC group to levels close to those in the Sham group (Figure 4A). We next analyzed the calcineurin/NFAT signaling pathway, an important signal transduction cascade underlying cardiac hypertrophy, by evaluating the expression of the target gene *Rcan1.4* (regulator of calcineurin). As shown in Figure 4B, *Rcan1.4* expression was significantly higher in the TAC group than in the Sham group, and was significantly lower in the TAC group treated with FICZ than in equivalent mice treated with vehicle.

Altogether, these data suggest that the calcineurin/NFAT axis could be a key contributor to the beneficial effects induced by FICZ in early cardiac hypertrophy development.

### 2.4. FICZ Treatment Prevents the Activation of Pro-Fibrotic Genes

**Figure 5:**
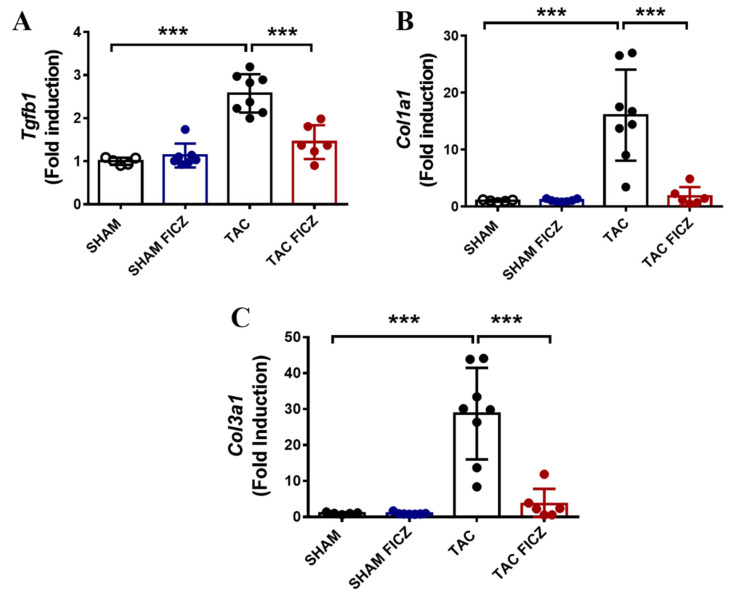
FICZ treatment prevents the increased expression of transforming growth factor beta 1 (*Tgfb1*), collagen type I alpha 1 (*Col1a1*), and collagen type III alpha 1 (*Col3a1*) in mice subjected to TAC surgery. Real-time PCR analysis of mRNA expression of *Tgfb1* (**A**), *Col1a1* (**B**), and *Col3a1* (**C**) normalized to that of the *Rplp0* housekeeping gene. Groups are: Sham (open circles) (*N* = 5), Sham FICZ (blue circles) (*N* = 7), TAC (black circles) (*N* = 8) and TAC FICZ (red circles) (*N* = 6). Data are expressed as mean ± SD. *** *p* < 0.001.

Fibrosis due to cardiac pressure overload is well documented [18]. Analysis of the expression of the pro-fibrotic genes transforming growth factor beta 1 (*Tgfb1*), collagen type I alpha 1 (*Col1a1*), and collagen type III alpha 1 (*Col3a1*) in the acute phase of TAC is shown in Figure 5A–C. Expression of these genes was significantly higher in the TAC than in the Sham group and, notably, significantly lower in the FICZ-treated TAC group than in vehicle-treated equivalent animals, indicating that FICZ attenuates the molecular activation of cardiac fibrosis in the early phase of pressure overload.

### 2.5. FICZ Treatment Suppresses Oxidative Stress via NRF2 Activation

**Figure 6:**
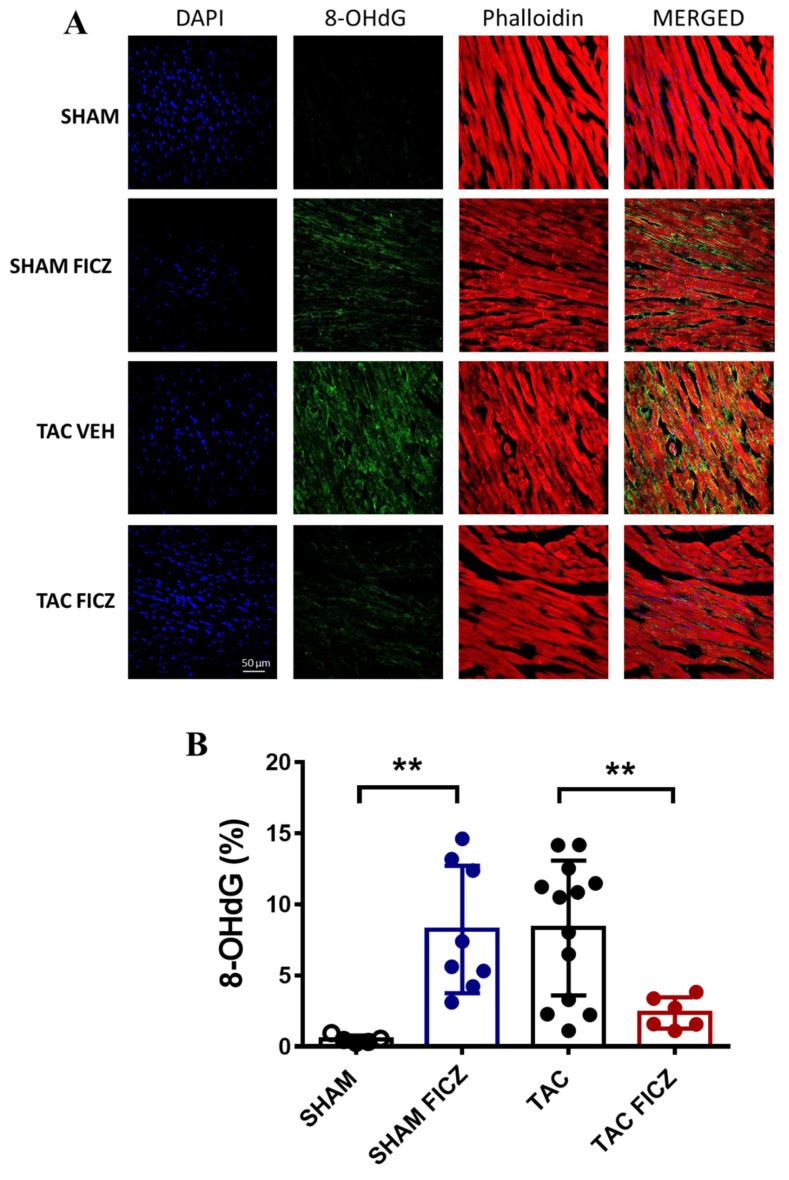
Cardiac staining of 8-hydroxy-2′-deoxyguanosine (8-OHdG) as a biomarker of DNA/RNA damage by oxidative stress: (**A**). Representative examples of immunofluorescent images obtained in all experimental groups. (**B**). Levels of 8-OHdG staining in the four groups of mice. Groups are: Sham (open circles) (*N* = 5), Sham FICZ (blue circles) (*N* = 8), TAC (black circles) (*N* = 13) and TAC FICZ (red circles) (*N* = 6). Data are expressed as mean ± SD. ** *p* < 0.01.

It is well known that cardiac pressure overload is associated with elevated oxidative stress in the heart. We thus analyzed the cardiac staining levels of 8-hydroxy-2′-deoxyguanosine (8-OHdG) as a biomarker of oxidative stress in the four groups of mice. Figure 6A shows representative examples of immunofluorescence images. As expected, oxidative stress was significantly higher in the acute phase of pressure overload in mice subjected to TAC surgery than in Sham-operated mice (Figure 6B). FICZ treatment induced a similar pro-oxidant effect in Sham mice, whereas an antioxidant effect was observed in TAC-operated mice, as evidenced by a lower level of 8-OHdG staining (Figure 6B).

**Figure 7:**
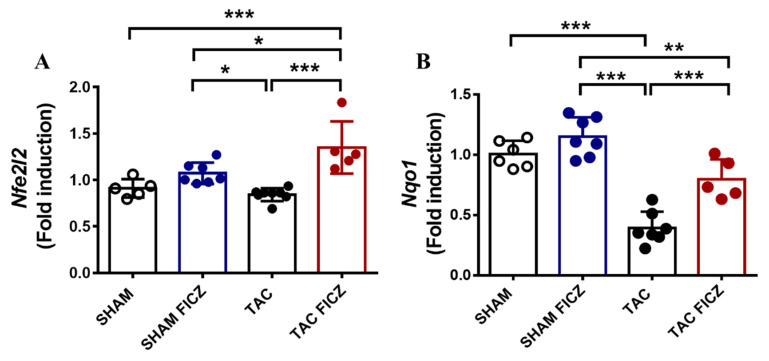
The antioxidant effect of FICZ on mice subjected to TAC surgery involves activation of the transcription factor nuclear factor erythroid derived 2-like 2 (*Nfe2l2*) and the antioxidant target gene NADPH/dehydrogenase quinone 1 (*Nqo1*). Real-time PCR analysis of *Nfe2l2* (**A**) and *Nqo1* (**B**), normalized to the housekeeping gene *Rplp0*. Groups are: Sham (open circles) (*N* = 5), Sham FICZ (blue circles) (*N* = 7), TAC (black circles) (*N* = 8) and TAC FICZ (red circles) (*N* = 5). Data are expressed as mean ± SD. *** *p* < 0.001; ** *p* < 0.01; * *p* < 0.05.

There is evidence that AhR engagement can trigger the activation of NRF2, a master regulator of the cellular antioxidant response [19]. To test the possible implication of NRF2 in the evident antioxidant effect of FICZ in mice subjected to TAC, we performed RT-PCR analysis of *Nfe2l2*, the gene encoding NRF2, and *Nqo1*, whose expression is stimulated by both NRF2 and AhR activation. As shown in Figure 7A, *Nfe2l2* expression was significantly higher in the TAC group treated with FICZ than in the Sham-vehicle and Sham-FICZ groups. Consistent with these results, the expression of *Nqo1* was lower in the TAC group than in the Sham group, and was significantly higher in the TAC group treated with FICZ than in equivalent mice treated with vehicle.

These results indicate that FICZ administration suppresses cardiac oxidative stress in the TAC mouse model, at least partly by activating NRF2 signaling.

## 3. Discussion

The present study provides the first evidence that the endogenous AhR ligand FICZ, one of the highest affinity agonists for AhR, improves adverse cardiac remodeling in the early phase of pressure overload in the murine heart. Specifically, we demonstrate that mice subjected to TAC surgery and treated with FICZ show reduced cardiac dilation and preserved EF compared with vehicle-treated TAC mice. In addition, FICZ treatment prevented cardiac hypertrophy and significantly reduced oxidative stress in the myocardium of mice subjected to 3 days of TAC, while an increase in oxidative stress was found in Sham mice treated with FICZ. FICZ treatment induced *Cyp1a1* expression several orders of magnitude higher than in vehicle-treated mice. This increase is likely the link between AhR activation and elevated ROS production, as *Cyp1a1* is part of the microsomal monooxygenase system that generates ROS even in the absence of substrates [20,21]. However, AhR activation may also favor the activation of the transcription factor NRF2 [22,23], which is considered as the master regulator of the cellular anti-oxidative response [19]. NRF2 induces the expression of several antioxidant enzymes including NQO1, which is systematically induced by various AhR ligands, including TCDD [24,25] and FICZ, in an NRF2-dependent manner. We found that the amelioration of oxidative stress evident in the TAC surgery group treated with FICZ was associated with a significant increase in *Nfe2l2* expression, and was accompanied by elevated *Nqo1* expression. Our results demonstrate an antioxidative response of AhR activation in the acute phase of pressure overload involving activation of the NRF2/NQO1 pathway. By contrast, the increase in oxidative stress found in Sham mice treated with FICZ or in non-treated TAC mice was not associated with changes in *Nfe2l2* mRNA expression. These findings support the idea that in a physiological context AhR activation triggers a pro-oxidant state that is not associated with deleterious cardiac remodeling or cardiac dysfunction. However, in a pathological context, in which oxidative stress is already present, activation of the NRF2/NQO1 pathway mediated by AhR engagement can act as a compensatory mechanism to reduce oxidative damage occurring in the early phase of pressure overload.

The CMRI study demonstrated that FICZ treatment prevents the increase in LVM in the TAC group, which is consistent with the results for the postmortem HW and HW/TL ratio and fits well with the results for *Nppa* expression. Cardiac overload is known to activate the calcineurin/NFAT/RCAN1.4 pathway. Dephosphorylation of NFAT by calcineurin allows NFAT to enter the nucleus, where it promotes the expression of target genes involved in cardiac hypertrophy [26]. RCAN1.4 is a calcineurin regulatory protein that functions as an endogenous feedback inhibitor, protecting the cell from uncontrolled calcineurin activity [27]. Elevated expression of *Rcan1.4* has been associated with the development of deleterious cardiac hypertrophy in several experimental models [28]. FICZ treatment prevented the up-regulation of *Rcan1.4* after TAC, supporting the involvement of the calcineurin/NFAT pathway in the antihypertrophic effect of this AhR ligand. Nevertheless, a deeper understanding of the calcineurin/NFAT pathway will be necessary to unambiguously demonstrate its involvement and its modulation by FICZ in the early phase of TAC.

The role of the many different AhR ligands on cardiac hypertrophy is likely to be complex. For instance, Maayah et al. [29] showed in Wistar albino rats that the anti-oncogenic drug sunitinib, which is a receptor tyrosine kinase inhibitor, promotes the expression of hypertrophic genes including *Nppa* via AhR activation. A similar phenomenon has been observed in a study with the AhR xenobiotic agonists 3-methylcholanthrene and benzo (a) pyrene in Sprague Dawley rats [30]. Indeed, some xenobiotics and drugs have been reported to mediate hypertrophic adaption through AhR [31]. Despite these conflicting results, studies with AhR-knockout mice have shown that cardiac hypertrophy is a common finding in these animals [13,14,32].

Regarding the fibrotic response, the picture is clearer; although, the precise role of AhR remains controversial [33]. We found that FICZ administration reduced the expression of *Tgfb1*, considered as a master regulator of fibrosis in the myocardium [34]. Among several mechanisms, inflammatory proteases including matrix metalloproteinases (MMPs) convert the stored latent form of TGF-β1 into an active form [35,36], which represses the synthesis of pro-inflammatory cytokines and MMPs and simultaneously promotes myofibroblast differentiation [37,38]. Numerous studies have associated fibrosis in AhR-knockout mice with a high level of *Tgfb1* expression [38], and several reports corroborate a reduction in the expression of *Tgfb1* mediated by AhR in different settings [39,40,41]. Thus, there appears to be a mutually negative interaction between the AhR and TGF-β1 pathways. Mechanistically, TGF-β1 signals canonically by mediating the phosphorylation of SMAD2 and SMAD3 proteins, which form a complex with SMAD4. This triple complex translocates to the nucleus, where SMAD3 binds to promoters of genes encoding collagen I and III α chains or the MMP inhibitor [34]. Activation of AhR interrupts this pathway by preventing the translocation of the SMAD complex to the nucleus [38]. This might explain the reduced expression of *Col1a1* and *Col3a1* in FICZ-treated TAC mice. Monteleone et al. [42] investigated the expression of *Col1a1* and *Col3a1* in intestinal fibroblasts from patients with Crohn’s disease, finding that the expression and secretion of *Col1a1* and *Col3a1* were elevated in the presence of TGF-β1, and was reduced by the addition of FICZ. They also found in a mouse model of intestinal fibrosis that FICZ administration decreased collagen production compared with control animals. Based on this, the authors concluded that AhR is a negative regulator of pro-fibrotic signals in the gut. In addition to regulating the gene expression of collagen α chains, AhR engagement by FICZ was reported to promote the expression of MMP1 collagenase, which degrades collagen in the extracellular matrix [38,43]. The ability of FICZ to attenuate pulmonary fibrosis by modulating the immune system via AhR has also been reported [23]. Accordingly, the antifibrotic effect found in the present study is consistent with much of the existing knowledge about AhR in other fields.

In conclusion, our study demonstrates that FICZ treatment at the onset of pressure overload-induced HF in the mouse is cardioprotective by exerting antihypertrophic, antifibrotic, and antioxidant effects.

## 4. Material and Methods

### 4.1. Animals and Experimental Protocol

All animal care and experimental procedures followed the guidelines for ethical care of experimental animals of the European Union (2010/63/EU) and were approved by the Bioethical Committee of the Consejo Superior de Investigaciones Científicas (Proex 184/17). Animal studies complied with the ARRIVE guidelines [44,45]. Male C57BL/6J mice (24–29 g, 10 weeks of age) were used in all experiments. Mice were bred and housed under specific pathogen-free conditions in the Experimental Animal Center of the Biomedical Research Institute “Alberto Sols” CSIC-UAM, Madrid, Spain. Animals were maintained at controlled temperature (23–25 °C) on a 12 h light/dark cycle with *ad libitum* access to water and a standard diet. The animal cages (Polysulfone type SII, Techniplast, Buguggiate, Italy) were 553 cm^2^, and animals were housed with a maximum of four mice per cage.

### 4.2. Transverse Aortic Constriction

Mice were anesthetized by intraperitoneal (i.p.) injection of a mixture of ketamine (100 mg kg^−1^) and xylazine (10 mg kg^−1^). Loss of withdrawal reflex was assessed before surgery. Mice were then intubated and the transverse aorta was constricted (TAC group) as described [46]. In the Sham group, trans-sternal thoracotomy was performed but the aorta was not ligated. Mice were given subcutaneous buprenorphine (1 mg kg^−1^) for pain relief after the surgery. A heated pad was used during surgery and recovery to maintain body temperature.

### 4.3. Study Design

**Figure 8:**
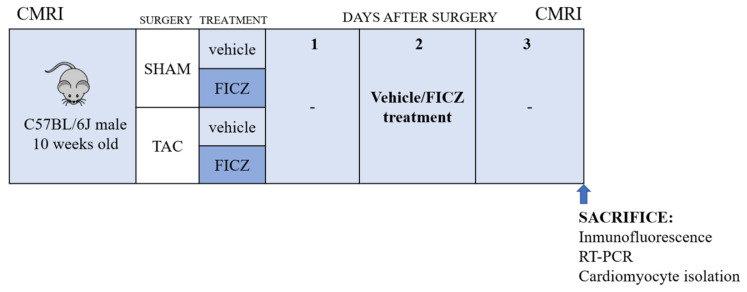
Scheme of the study design.

Animals were randomized into the following four groups: Sham-treated with vehicle or with FICZ and TAC-treated with vehicle or with FICZ (Figure 8). Treatment with FICZ (i.p., 5 mg kg^−1^) or with vehicle (dimethylsulfoxide) was initiated 5 min after Sham or TAC surgery and was repeated 2 days after surgery. CMRI was performed on 8 animals in each group before and 3 days after surgery. Subsequently, animals were anesthetized with isoflurane, and hearts were excised, weighed, and prepared for biochemical and immunofluorescence studies. In some experiments, hearts were retrogradely perfused through the aorta to isolate left ventricular myocytes, as reported [46]. Cardiomyocyte images were acquired using a Nikon 90i microscope and Nis Elements 3.22 software. The surface area of cardiomyocytes was quantified using ImageJ (NIH) (RRID:SCR_003070). A single investigator blinded to the experimental groups performed the analysis.

### 4.4. Cardiac Magnetic Resonance Imaging

CMRI was carried out on a 7.0 Tesla MR system (Bruker Pharmascan, Bruker, Ettlingen, Germany) in the Biomedical Research Institute “Alberto Sols” CSIC-UAM. The procedure was the same as described previously [46]. Briefly, mice were anesthetized with an isoflurane and oxygen mixture (2% in 1 L min^−1^ for induction and 1.5% during acquisition), and temperature, heart, and respiratory rates were monitored during the study using the 1025 SAM monitoring and gating system (SA Instruments, Inc., New York, NY, USA). After position adjustment, 6 to 12 slices were acquired to cover the entire heart. Each slice consisted of 20 gated time frames synchronized with the cardiac cycle. The acquired data were zero-filled to achieve a reconstructed matrix size of 256 × 256.

For image analysis, we used SEGMENT v2.0 R5642 (http://segment.heiberg.se, accessed on 26 April 2022). Six slices were selected from each heart and were analyzed by manual segmentation of left ventricular endocardial and epicardial borders in all the image frames. After segmentation of the images, SEGMENT was used to calculate the following functional parameters: LVEDV (μL), LVESV (μL), EF (%), and LVM (mg).

### 4.5. Immunofluorescence Analysis

Freshly dissected heart tissues were fixed with 10% formalin for 24 h at room temperature (RT), cryoprotected in 30% sucrose (Merck, Madrid, Spain) at 4 °C until they were completely submerged, and then frozen at −80 °C in Tissue-Tek O.C.T. (Sakura Finetek Europe B.V., Alphen aan den Rijn, The Netherlands). Cryostat sections (10 µm thickness) were fixed with 4% paraformaldehyde for 20 min at RT and incubated with a permeabilizing/protein blocking solution (5% bovine serum albumin + 10% goat serum + 0.3% Triton X-100) for 1 h at RT. Afterward, tissue sections were incubated with a monoclonal anti-8-OHdG primary antibody (1:500; Abcam, Cambridge, UK; #62623) overnight at 4 °C. Sections incubated in the absence of the primary antibody were used as negative controls. Tissue sections were washed with phosphate-buffered saline and incubated with a goat anti-mouse IgG (H + L) Highly Cross-Adsorbed Secondary Antibody, Alexa Fluor^®^ 488 (1:500; ThermoFisher Scientific, Waltham, MA, USA; #A11029) for 120 min at RT. Then, F-actin fibers were stained using Alexa Fluor™ 546 Phalloidin (1:40 Thermo Fisher Scientific; #A22283) for 20 min at RT and nuclei with DAPI (1:500; Roche, Indianapolis, IN; #10236276001) for 12 min at RT. Finally, all sections were mounted with ProLong™ Diamond Antifade Mountant (ThermoFisher Scientific; #P36970) and were examined with a Zeiss LSM 710 laser scanning confocal microscope, with a 25× objective (Carl Zeiss AG, Oberkochen, Germany).

Quantification of oxidative stress was performed using ImageJ (NIH) (RRID:SCR_003070) and expressed as the percentage of the area marked with the anti-8-OHdG antibody divided by the total amount of tissue measured by Phalloidin staining.

### 4.6. RNA Isolation and RT-PCR

Left ventricle tissue excised from mice of each experimental group was stored for 24 h in RNAlater solution (Qiagen, Hilden, Germany) at 4 °C and then kept at −80 °C until use. Total RNA was extracted using the RNeasy Mini Kit on a QIAcube robotic workstation (Qiagen) and was quantified using a NanoDrop spectrophotometer (NanoDrop Technologies, Wilmington, DE, USA). The High-Capacity cDNA Reverse Transcription Kit (Applied Biosystems, Foster City, CA, USA) was used for retrotranscription and RT-PCR was performed on an ABI 7900HT Fast RT-PCR platform (Applied Biosystems). The *Rplp0* gene (*36B4*) was used as a housekeeping gene for fold-induction calculations using the ∆∆Ct method.

The primers used are detailed below. All were purchased from Invitrogen (Carlsbad, CA):

*Cyp1a1* forward 5′-GGTTAACCATGACCGGGAACT-3′ and reverse 5′-TGCCCAAA CCAAAGAGAGTGA-3′; *Nppa* forward 5′-ATTGACAGGATTGGAGCCCAGAGT-3′ and reverse 5′-TGACACACCACAAGGGCTTAGGAT-3′; *Rcan1.4* forward 5′-GA GCGAGT CGTTCGTTAAGC-3′ and reverse 5′-GCCACACAAGCAATCAGGGA-3′; *Col1a1* forward 5′-AATGGCACGGCTGTCTGCGA-3′ and reverse 5′-AGCACTCGCC CTCCCGTCTT-3′; *Col3a1* forward 5′-CTGTAACATGGAAACTGGGGAAA-3′ and reverse 5′-CCATAGCTGAACTGAAAACCACC-3′; *Tgfb1* forward 5′-CTGCTGA CCCCCACTGATAC-3′ and reverse 5′-AGCCCTGTATTCCGTCTCCT-3′; *Nfe2l2* forward 5′-TAGATGACCATGAGTCGCTTGC-3′ and reverse 5′-GCCAAACTTGCT CCATGTCC-3′; *Nqo1* forward 5′-GGTAGCGGCTCCATGTACTC-3′ and reverse 5′-CATCCTTCCAGGATCTGCAT-3′; *Rplp0* forward 5′-AGATGCAGCAGATCCGC AT-3′ and reverse 5′-GTTCTTGCCCATCAGCACC-3′.

### 4.7. Statistical Analysis

Data are expressed as mean ± standard error of the mean (SEM) in CMRI graphs and mean ± standard deviation (SD) when individual values are included in the plot. One-way ANOVA was used to compare significance among groups. When ANOVA produced a significant value of F (*p* < 0.05) and there was no significant variance inhomogeneity, Bonferroni’s post hoc multicomparison analysis was applied. All tests were two-tailed and *p*-values < 0.05 were considered statistically significant. Statistical analysis and graph plotting were performed using GraphPad Prism v.9.0 (GraphPad Software Inc., La Jolla, CA, USA).

## Data Availability

The data that support the findings of this study are available from the corresponding authors upon reasonable request.
